# Inclusive Fitness Theorizing Invokes Phenomena That Are Not Relevant for the Evolution of Eusociality

**DOI:** 10.1371/journal.pbio.1002134

**Published:** 2015-04-24

**Authors:** Martin A. Nowak, Benjamin Allen

**Affiliations:** 1 Program for Evolutionary Dynamics, Harvard University, Cambridge, Massachusetts, United States of America; 2 Department of Organismic and Evolutionary Biology, Department of Mathematics, Harvard University, Cambridge; 3 Department of Mathematics, Emmanuel College, Boston, Massachusetts, United States of America; 4 Center for Mathematical Sciences and Applications, Department of Mathematics, Harvard University, Cambridge, Massachusetts, United States of America

## Abstract

In this Formal Comment, the authors challenge the claims of a recent theoretical study that genetic relatedness is important in the evolution of eusociality.

Inclusive fitness, long regarded as an important concept in sociobiology, was shown by Nowak, Tarnita, and Wilson (NTW) [1] to be of limited value for understanding the evolution of social behavior. Attempts to rebut these findings have relied on dubious mathematics and verbal misrepresentations of the key results. Similarly, Liao, Rong, and Queller (LRQ) [2] present a distorted view of NTW, introduce models for hypothetical phenomena that have no relevance for evolution of eusociality, and fail to analyze their own models with inclusive fitness theory.

What are the main points of NTW? (i) Inclusive fitness, as defined by Hamilton and as used in all meaningful papers on the subject ever since, is a limited concept, which does not exist for the majority of evolutionary processes. Consequently, whenever biologists use the term “inclusive fitness” to refer to an actual population, they are almost certainly in a situation in which that quantity does not exist. (ii) There is a powerful mathematical theory of evolution, which can explain social behavior without using inclusive fitness. (iii) Eusociality can arise by a simple mutation that causes some offspring to stay at their mother’s nest. The success of eusociality depends on demographic and ecological parameters, while relatedness is the same for all species under primary consideration.

What was the main discussion since NTW? Some theorists have argued that inclusive fitness can always be calculated using a method based on linear regression [3], but Allen et al. [4] have demonstrated that this method (i) cannot make predictions and (ii) mistakes correlation for causation [5]. The interpretation of regression coefficients as “benefit” and “cost” in this method is unjustified, and departs from the legitimate uses of regression in quantitative genetics. Another recent finding is that inclusive fitness calculations, even when they are possible, can give the wrong result as to the direction of natural selection [6].

In attempting to uphold a discussion of inclusive fitness, LRQ perform three extensions of the eusociality model of NTW.

In their Model 1, LRQ introduce mixing of workers between nests. Three interpretations of this mixing are offered: (i) queens take over other colonies (nest usurpation), (ii) workers migrate between colonies, or (iii) queens lay eggs into other nests. LRQ’s first interpretation is incompatible with their own mathematical model, which does not contain any terms for queens taking over nests.

In the second interpretation, if a solitary mother reproduces, then the following sequence of steps is executed: (i) with probability *r* (which is interpreted as "relatedness"), the new individual does not become a migrant but leaves and starts its own colony; (ii) with probability 1 − *r*, the new individual becomes a migrant; now one of two things can happen: (iii) with probability *f*_*s*_ the individual is replaced by a solitary one that starts its own colony; or (iv) with probability *f*_*e*_ the individual is replaced by a eusocial one that goes back to the colony from which the migrant emerged; there the individual can either (v) stay with probability *q* and thus increase the size of the colony headed by the solitary mother or (vi) with probability 1 − *q*, the now eusocial individual leaves and starts its own eusocial colony. The model does not seem to describe a plausible biological scenario.

Moreover, the migrant pool has the following strange property: as soon as a colony enters an individual into the migrant pool, that colony gets an individual back from the migrant pool. In contrast, if an individual dies or leaves a colony to form its own nest, then the size of the colony decreases. In LRQ’s model, none of the migrant workers ever have a chance to take over the nest.

For the origin of eusociality, LRQ’s model assumes a presocial species in which a fraction of offspring migrate to new nests, but subsequently all offspring leave to start nests of their own, rendering the initial migration useless. Eusociality arises via an extraordinary mutation that reduces the second "leaving" action while having no effect on the initial "migration" action.

For interpreting the model in terms of mothers laying eggs into other nests, LRQ assume that as soon as a mother lays an egg into another nest, she receives an egg into her own nest. The magnitude of egg swapping that occurs in their model is astonishing: if r < 0.5 (as in their Fig 1) then the dominant mode of reproduction is that a mother lays her own eggs into other nests and, in return, raises the eggs of other mothers. It is hard to imagine that those phenomena occur in nature, let alone are relevant for the origin of eusociality.

**Fig 1:**
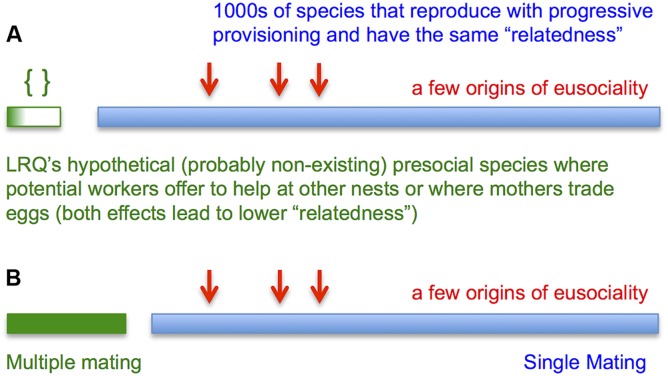
Does “relatedness” cause evolution of eusociality? (a) There are thousands of species that reproduce with progressive provisioning without trading workers or eggs. They all have the same “relatedness.” Yet only a small number of them make a transition to eusociality. The main question is: which ones make the transition and under what conditions? This question was studied by NTW. LRQ’s first model adds that imaginary (and probably non-existing) presocial species with hypothetical trading of workers or eggs are less likely to evolve eusociality. (b) A more meaningful question, not studied by LRQ, would have been whether eusociality is more likely to evolve if the queen mates once or several times [15]. But again a fundamental question here is: what is the condition for the rare emergence of eusociality in species with single mating? This question, never addressed by inclusive fitness theory, was studied by NTW. Since all those species have the same relatedness between mothers and offspring, but only very few of them evolve eusociality, “relatedness” is not the answer.

LRQ cite two studies [7,8] justifying their biological assumptions, but neither study lends actual support. Field [7] reports only a small percentage of eggs being laid into other nests in three species (2%,7%, and 0%–15%), while none of the other phenomena investigated in Field’s paper (such as nest usurpation) are compatible with LRQ’s mathematical equations. Abbot et al. [8] describe social parasitism among aphids, in which reproductive individuals invade another gall to reproduce there; again this phenomenon is not compatible with LRQ’s equations, in which only a single resident queen reproduces in each nest and is never replaced.

Why do LRQ investigate such models? They present NTW as saying relatedness does not matter in general, but this is incorrect. Instead NTW write, "Relatedness does not drive the evolution of eusociality. We can use our model to study the fate of eusocial alleles that arise in thousands of different presocial species with haplodiploid genetics and progressive provisioning. In some of those species eusociality might evolve, while in others it does not. Whether or not eusociality evolves depends on the demographic parameters of the queen (…), but not on relatedness. The relatedness parameters would be the same for all species under consideration" (Fig 1).

NTW have presented a simple model for the origin of eusociality, which requires no variation in relatedness. LRQ try to force variation of relatedness into this model unnaturally and for no biological reason.

LRQ’s approach also highlights a major problem of inclusive fitness thinking: “relatedness” is treated as an independent causal agent, such that one way of varying relatedness is equivalent to any other. This problem was addressed by NTW, who wrote, “It is possible to consider situations where all measures of relatedness are identical, yet cooperation is favoured in one case, but not in the other. Conversely, two populations can have relatedness measures on the opposite ends of the spectrum and yet both structures are equally unable to support evolution of cooperation. Hence, relatedness measurements without a meaningful theory are difficult to interpret." The relatedness discussions of NTW are more sophisticated than what is presented by LRQ.

“Relatedness” is often used as inclusive-fitness jargon for population structure, giving the wrong impression that all aspects of population structure can be described by a single quantity. Decades of research [9–13] have shown that the effects of population structure on evolutionary dynamics are more intricate than such a view would suggest.

LRQ find that their Model 1 makes it harder for eusociality to emerge than the original model of NTW. But this effect can be understood immediately without recourse to "relatedness": solitary mothers use the mixing pool to gain eusocial offspring that help them reproduce, while eusocial mothers receive solitary offspring who do not help.

LRQ’s Model 2, examining maternal control, is based on the following assumptions. Solitary mothers force eusocial migrants, who offer to help, away from their nest. Eusocial mothers receive solitary migrants, conscript some of them to help, but force others to leave and build their own solitary nests. According to this model, eusociality originates as follows: in the solitary ancestor species, there are already potential workers who move between nests and offer to help, but they are not admitted. The step to eusociality is a mutation in which a mother allows those workers to join her nest. Again there does not seem to be any biological evidence in favor of such a model.

Model 2 is directed at NTW’s remark that workers can be seen as robots. But this remark indicates the difference in evolutionary dynamics when interactions occur between mother and offspring staying together as opposed to independent agents coming together [14]. Everyone understands that mutations expressed in queens or workers can lead to different population genetic models.

LRQs' finding that eusociality evolves more readily in Model 2 is easily explained: eusocial mothers conscript migrants, whereas solitary mothers force them away. Now the migrant pool helps the eusocial mothers. Any conflict can be understood without invoking relatedness: eusocial mothers try to conscript solitary workers, but solitary individuals try to build their own nests.

A feature of Model 2 is that eusociality evolves even for zero relatedness. Thus, LRQ actually propose in their Fig 1 that relatedness is neither necessary nor sufficient for the evolution of eusociality.

In Model 3, LRQ abandon migration and compare two different fitness functions for the original model of NTW, which is a useful investigation in principle. LRQ make great efforts to minimize the fitness advantage that is needed for eusociality to emerge. They overlook, however, that the formula for the minimum threshold is already provided by NTW (see SI eq. 55). For obtaining the minimum threshold, the maximum advantage has to arise for the smallest colony size (*m* = 2), and there is no worker mortality. Then the condition is

$$b-2d>2(b_{0}-d_{0}).$$

Here *b* and *d* are the reproductive and death rate of the queen if she has at least one helper; *b*_0_ and *d*_0_ are the corresponding quantities when she is alone. None of the numerical values obtained by LRQ are below the threshold given above. Hence in their Model 3, LRQ do not contradict any result of NTW, but only provide a numerical confirmation.

In their attempt to uphold inclusive fitness, LRQ do not offer any inclusive fitness calculation for their first two models, where “relatedness” *r* varies. We are neither told what the inclusive fitness is for solitary or eusocial individuals, nor what relatedness is (in terms of identity by descent) for any pair of individuals. LRQ only try to calculate a version of Hamilton’s rule for *r* = 1, but this calculation is incorrect: offspring who stay and leave are treated the same—ignoring differences in reproductive value—and there is no consideration of death terms.

NTW’s criticism of inclusive fitness is deeper than what is presented by LRQ, and contrary to what LRQ suggest, the mathematical facts proven by NTW have never been answered or negated by inclusive fitness proponents. LRQ’s paper demonstrates how inclusive fitness theorizing becomes an end in itself, which distracts from the biological questions at hand. In contrast, the mathematical theory of evolution is clear and powerful and shows that the concept of inclusive fitness is not needed to understand any phenomenon in evolutionary biology.
